# The Effects of Maternal Obesity on Porcine Placental Efficiency and Proteome

**DOI:** 10.3390/ani9080546

**Published:** 2019-08-12

**Authors:** Ji-Wei Li, Jian Hu, Ming Wei, Ying-Ying Guo, Pei-Shi Yan

**Affiliations:** College of Animal Science and Technology, Nanjing Agricultural University, Nanjing 210095, China

**Keywords:** pig, placenta, maternal obesity, isobaric tags for relative and absolute quantification, proteomics

## Abstract

**Simple Summary:**

The placenta plays an important role in the growth and development of a fetus and in maintaining a healthy pregnancy. The placenta is also sensitive to suboptimal intrauterine conditions, such as maternal obesity. Although previous studies have indicated that maternal obesity is associated with placental dysfunction, we are still far from elucidating the molecular mechanisms underlying this phenomenon. In this study, proteomic technique was employed to compare the proteomic profiles of the placentas of sows with different backfat thicknesses. We revealed that excessive backfat of sow is associated with abnormal carbohydrate and lipid metabolism, mitochondrial dysfunction, and increased oxidative stress and inflammation in the placenta. Our results promote the understanding of decreased placental efficiency induced by maternal obesity.

**Abstract:**

Maternal obesity is associated with impaired maternal metabolism and affects the developmental programming of the fetus. The placenta is dysfunctional when exposed to an obese intrauterine environment and can transduce and mediate detrimental maternal impacts to the fetus through mechanisms that remain largely unknown. The main objective of this study was to investigate the effects of maternal obesity on the porcine placental proteome and to analyze the deregulated proteins and potential pathways predicted to be disturbed in obese placentas, using sows with high backfat as a model of obese pregnancy. The sows were divided into two groups based on their backfat thickness: normal backfat (NBF, 17–22 mm; n = 30) and high backfat (HBF, ≥23 mm; n = 30) as the maternal obesity group. The placental tissues used for the proteomic and biochemical analyses were obtained through vaginal delivery, and the maternal blood samples used to determine the metabolic parameters were collected at day 107 of pregnancy. Our study demonstrated that HBF sows had significantly decreased placental efficiency, increased plasma-free fatty acids and triglyceride levels, and increased proinflammatory cytokines plasma levels (*p* < 0.05). HBF placentas had significantly higher malondialdehyde level, lower total antioxidant capacity and antioxidase activity, increased triglyceride content and enhanced proinflammatory tumor necrosis factor- α (TNF-α) and interleukin-6 (IL-6) contents (*p* < 0.05). Among the 4652 proteins identified using the proteomic method, 343 were quantified as differentially abundant proteins, which were involved in many vital biological processes. Based on our bioinformatic and placental biochemical analyses, we concluded that maternal obesity is associated with abnormal carbohydrate and lipid metabolism, mitochondrial dysfunction, decreased steroid hormone biosynthesis, and increased oxidative stress and inflammation in the placenta. The results of this study are undoubtedly valuable to other researchers.

## 1. Introduction

The prevalence of maternal obesity has increased dramatically [1] and is associated with impaired fecundity in humans as well as in some animals, such as pigs [2,3]. Clinical research has demonstrated that an aberrant intrauterine environment induced by maternal obesity is related to increased obstetrical complications in mothers and infants [4,5]. Similarly, in pigs, a significant negative correlation has been observed between the backfat thickness of sows and their subsequent reproductive performance [3,6,7]. Obese pregnancy provokes abnormal maternal metabolism, including the dysregulation of cytokines and obesity-associated metabolic hormones, which leads to placental dysfunction and an adverse pregnancy outcome [2].

The placenta plays a crucial role in the regulation of substance exchange between maternal and fetal circulation and hormone production, and is sensitive to suboptimal intrauterine conditions, such as maternal obesity. Recent evidences demonstrate that maternal obesity results in lipotoxicity characterized by ectopic fat accumulation [8], augmented inflammatory responses and macrophage accumulation [9], and elevated nitrative and oxidative stress [10,11] in the human placenta, which may promote impaired trophoblast functions and alterations in placental nutrient transport [12]. In our previous study, increased triglyceride (TG) and free fatty acids (FFA) levels were consistently observed in epitheliochorial placentas from obese Landrace sows [13]. The challenge of intrauterine stress associated with maternal obesity is that it can disrupt normal placental function, and thus impact fetal growth and development through mechanisms that remain largely unknown.

The proteomic technique has been deemed a powerful tool for unveiling complex changes during disease processes. In the present study, we applied isobaric tags for relative and absolute quantification (iTRAQ) coupled with two-dimensional liquid chromatography and tandem mass spectrometry (2DLC-MS/MS) to compare the quantitative changes in the proteome of the placenta in response to obese pregnancy, using sows with high backfat as a model of obese pregnancy [14]. The main objective of this study was to identify the differentially abundant proteins (DAPs) in obese sows’ full-term placentas and to analyze the deregulated proteins and potential pathways predicted to be disturbed in obese placentas.

## 2. Materials and Methods

### 2.1. Animals and Study Design

All animal procedures used in the present study were approved by the Laboratory Animal Care Committee of Nanjing Agricultural University (Authorization number: SYXK [Su] 2011-0056). A retrospective cohort of multiparous Yorkshire sows with diverse backfat thicknesses at the time of mating was selected on the 107th day of pregnancy in this study. The total number of selected sows was 60 (parity = 3), and these were divided into two groups according to their backfat thickness. Sows with backfat between 17 and 22 mm were defined as having normal backfat (NBF; n = 30), and those with backfat ≥23 mm were deemed as having high backfat (HBF; n = 30). All sows were individually housed, provided water freely and fed twice daily with the same pellet feed (15.5% crude protein, 13.1 MJ ME/kg) during pregnancy.

### 2.2. Data Collection and Sampling

The backfat thickness of the P2 position (Figure S1) at mating and farrowing was measured using A-mode ultrasonography (Renco). Live-born and stillborn piglets were individually weighed and counted immediately postpartum. On the 107th day of pregnancy, maternal fasting blood samples were collected from the ear vein and centrifuged for 15 min at 3500× *g*. The corresponding plasma was stored at −80 °C for further analysis. Each placenta was weighed immediately following delivery to calculate the placental efficiency as a ratio between the piglet’s weight and the placental weight [15]. In order to match individual piglets with their placentas, when each piglet was farrowed, we tied its umbilical cord using a long suture with a numbered tag to match the birth order of the piglet (classical Wilson’s method [16]). Thus, even though the umbilical cord retracts into the birth canal, we can also ensure that collected placentas belong to their respective piglets. About 10 g of placental villous tissue, which came from five randomized sampling points in one placenta, was collected and stored at −80 °C for further analysis. 

### 2.3. Plasma Analysis

Plasma TG, FFA, total cholesterol (CHOL), high-density lipoproteins (HDL) and low-density lipoproteins (LDL) were individually measured using enzymatic reagents (Diasys Diagnostic Systems, Holzheim, Germany). Glucose was measured using the oxidase method (Yellow Springs, OH, USA). Porcine ELISA kits were used to assay plasma levels of IL-6 and TNF-α (R&D Systems, Minneapolis, MN, USA). All samples were measured in triplicate in a single assay according to the manufacturers’ instructions.

### 2.4. Biochemical Analysis of Placental Tissue

Placental tissues were homogenized using a tissue tearer (IKA, Shanghai, China) in lysis buffer as described earlier [10] and centrifuged at 10,000× *g* for 10 min at 4 °C. The supernatant was used to assay the contents of TNF-α, IL-6 (R&D Systems), CRP (Elabscience, Wuhan, China), pentraxin-3 (MyBioSource, San Diego, CA, USA) and ITIH4 (PigCHAMP Pro Europa, Spain) via Porcine ELISA Kits. The TG and malondialdehyde (MDA) levels of the placental homogenates were determined using enzymatic reagents (Diasys Diagnostic Systems, Holzheim, Germany) and the MDA Assay Kit (Beyotime, Shanghai, China). Placental homogenates were assayed for total antioxidant capacity (T-AOC), superoxide dismutase (SOD), catalase (CAT) and glutathione peroxidase (GSH-PX) activity (Cayman Chemical, Ann Arbor, MI, USA), according to the manufacturer’s instructions. All samples were normalized according to placental protein concentrations quantified using the BCA Protein Assay Kit (Thermo Scientific, Rockford, IL, USA).

### 2.5. Placental Protein Extraction for Proteomics

Placental samples were pulverized under liquid nitrogen and protein extraction was performed as described before [17]. In the proteomic experiment, we adopted a single pooling strategy which has been used widely to reduce the effect of biological variation in the proteomic experimental design [18,19,20]. In detail, ten individual placental samples, which were randomly selected from ten sows from each backfat group, were pooled as a biological replicate. Each backfat group included three pooled biological replicates (NBF1, NBF2, NBF3 and HBF1, HBF2, HBF3) (Figure S2).

### 2.6. iTRAQ Labelling and SCX Fractionation

For each mixed sample, 100 μg of protein was digested with Trypsin (Promega, Madison, WI, USA) with the ratio of protein:trypsin = 30:1 at 37 °C for 16 h. Subsequently, peptides were dried using vacuum centrifugation and re-dissolved in 0.5 M Tetraethylammonium bromide. The tryptic peptides were labelled with the iTRAQ tags as follows: NBF1 (tag 114), NBF2 (tag 116), NBF3 (tag 118), HBF1 (tag 117), HBF2 (tag 119) and HBF3 (tag 121). Finally, the labelled peptides were mixed and vacuum-dried.

iTRAQ-labelled peptides were fractionated by SCX chromatography using the LC-20AB HPLC Pump system (Shimadzu, Kyoto, Japan). The dried peptide mixtures were reconstituted with 4 mL buffer A (25 mM NaH_2_PO_4_ in 25% ACN, pH 2.7) and loaded onto a 4.6 × 250 mm Ultremex SCX column containing 5-μm particles. The peptides were eluted at a flow rate of 1mL/min with a gradient of buffer A for 10 min, 5–60% buffer B (25mM NaH_2_PO_4_, 1 M KCl in 25% ACN, pH 2.7) for 27 min and 60–100% buffer B for 1 min. Elution was monitored by absorbance at 214 nm, and fractions were collected every 1 min. The eluted peptides were pooled into 20 fractions, desalted with a Strata X C18 column and vacuum-dried.

### 2.7. LC-ESI-MS/MS Analysis Based on Triple TOF 5600

Each dried fraction was resuspended in Buffer A (5% ACN; 0.1% FA), and 10 μL of the peptide solution (0.5 μg/μL) was loaded onto an LC-20AD nano-HPLC column (Shimadzu, Kyoto, Japan) at 8 μL/min for 4 min. Then, the 35-min gradient was run at 300 nL/min starting from 2% to 35% Buffer B (95% CAN; 0.1% FA), followed by a 7-min linear gradient to 80% Buffer B, maintained at 80% Buffer B for 4 min, and finally, returned to 5% Buffer B in 1 min. MS data was acquired using a Triple TOF 5600 System (AB SCIEX, Concord, Canada). The parameters of the MS/MS included the following: ion spray voltage of 2.5 kV, curtain gas of 30 psi, nebulizer gas of 15 psi, and an interface heater temperature of 150 °C. For the information dependent acquisition, survey scans were acquired in 250 ms and as many as 30 production scans were collected if a threshold of 120 counts per second with a 2+ to 5+ charge-state was exceeded. A sweeping collision at an energy setting of 35 ± 5 eV coupled with the iTRAQ adjusted rolling collision energy was applied to all precursor ions for collision-induced dissociation. Dynamic exclusion was set for 1/2 of peak width (15 s), and then the precursor was refreshed off the exclusion list.

### 2.8. Protein Identification and Quantification

The raw mass data were converted using Proteome Discover 1.3, and protein identification was performed using Mascot Software 2.3.02 (Matrix Science, London, UK) against the Uniprot Sus scrofa database (33,785 sequences). For the protein identification, the following options were used: enzyme = trypsin; missed cleavage = 1; peptide mass tolerance = ±0.05 Da; fragmented ions tolerance = ±0.1 Da; fixed modification = Carbamidomethyl (C), iTRAQ 8 plex (N-term) and iTRAQ 8 plex (K); potential variable modification = Gln→pyro-Glu (N-term Q), Oxidation (M) and Deamidation (NQ). The protein with at least one unique peptide, which had a significant score at the 95% confidence interval through the Mascot probability analysis, was counted as identified. The quantitative protein ratios were weighted and normalised by the median ratio in Mascot. The protein with three abundance ratios >1.2 (or < 0.83) and *p*-value < 0.05 was considered as DAP.

### 2.9. Bioinformatics Analysis

The biological process (BP), molecular function (MF) and cellular component (CC) of all the proteins identified by iTRAQ were annotated by Gene Ontology (GO, http://www.geneontology.org). Pathway analyses of identified proteins were performed using the Kyoto Encyclopedia of Genes and Genomes (KEGG, http://www.genome.jp/kegg/). The GO term and KEGG pathway enrichment analyses of the DAPs were conducted using a hypergeometric test. Protein-protein interaction (PPI) networks were built by the Search Tool for the Retrieval of Interacting Genes/Proteins (STRING 10.5). The PPIs with a combined (edge) score of greater than 0.7 (high confidence) were used for further network analysis. Cytoscape 3.4.0 was used to visualize the interaction networks [21].

### 2.10. RNA Extraction and RT-qPCR

Total RNA was extracted from placental tissue from each group (30 placentas) using the TRIzol Reagent (Life technologies). One microgram of total RNA per sample was used for cDNA synthesis using PrimeScript RT Master Mix Kit (TaKaRa, Tokyo, Japan) and an ABI 2700 thermal cycler (ABI, Foster city, CA, USA) with the following procedure: 37 °C for 15 min, 85 °C for 5 s and 4 °C hold. Quantitative real-time PCR was performed using 10 μL of 2 × SYBR premix Ex Taq (TaKaRa, Tokyo, Japan), 2 μL of synthesized cDNA and gene-specific primers (Table S1) on the QuantStudio 7 Flex System (ABI, Foster city, CA, USA) with the following program: 95 °C for 30 s; 95 °C for 5 s, 60 °C for 30 s (40 Cycles); 95 °C for 15 s, 60 °C for 60 s, 95 °C for 15 s. Dissociation curve was added to verify product specificity. The expression level of each gene, relative to the reference gene (*GAPDH*), was calculated according to the 2^−ΔΔCt^ method.

### 2.11. Statistical Method

The reproductive performance parameters, blood indexes and placental biochemical parameters were compared between the two groups using the *t*-test procedure of SAS 9.4 (SAS Inst., Inc., Cary, NC, USA) as a complete randomized design, and *p* < 0.05 was considered statistically significant. Data are presented as mean ±SD.

## 3. Results

### 3.1. Characteristics of the Studied Sows

The reproductive performance parameters of the studied sows are presented in Table 1. As expected, backfat was significantly greater (about 7 mm thicker) at breeding and farrowing in the HBF group. The number of low body weight piglets (LBW, piglets with weight < 0.9 kg), percentage of stillborn piglets and within-litter variation of piglet weights were significantly greater in the HBF sows. On the other hand, the HBF sows had significantly lower average weight of live-born piglets than the NBF sows. The HBF sows had significantly lower placental efficiency. 

The plasma parameters of the studied sows are displayed in Table 1. Although there were no differences in the HDL, LDL, CHOL and glucose plasma levels, plasma TG and FFA levels increased considerably in the HBF sows. The HBF sows were associated with increased contents of the circulating IL-6 and TNF-α.

### 3.2. Antioxidant Status and TG and Inflammatory Cytokine Contents in the Placentas

**Figure 1:**
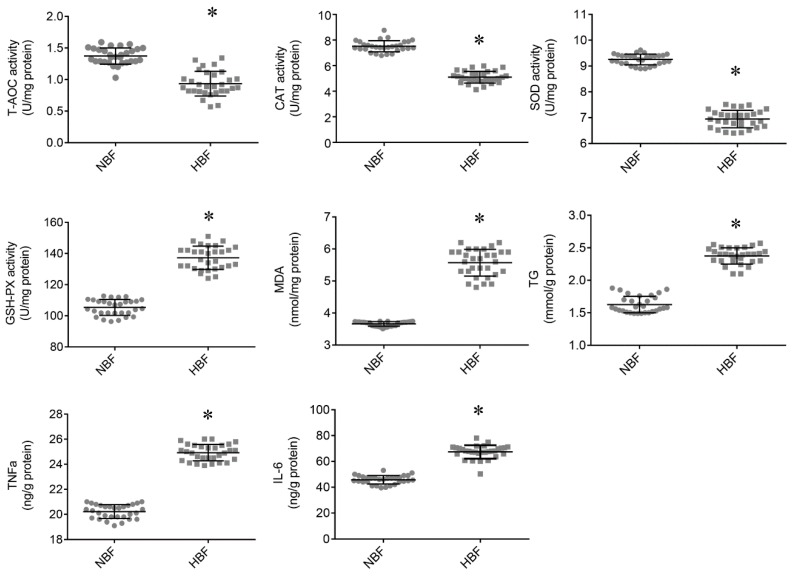
Antioxidant status, TG and inflammatory markers of full-term placentas. Abbreviations: SOD, superoxide dismutase; CAT, catalase; GSH-PX, glutathione peroxidase; T-AOC, total antioxidant capacity; MDA, malondialdehyde; TG, triglyceride. n = 30 biological replicates in each group. Values are presented as means ± SEM. * *p* < 0.05.

As shown in Figure 1, HBF placentas had significantly higher MDA level and lower T-AOC, CAT and SOD activities. However, the GSH-PX activity was significantly greater in the HBF placentas. In this study, we also observed that HBF placentas had significantly increased TG content. In addition, the HBF sows exhibited enhanced pro-inflammatory TNF-α and IL-6 contents in their placentas compared to the NBF sows. These results indicated that HBF placentas had increased ectopic fat accumulation, augmented inflammatory responses and elevated oxidative stress.

### 3.3. Identification and Quantification of the Placental Proteins

**Figure 2:**
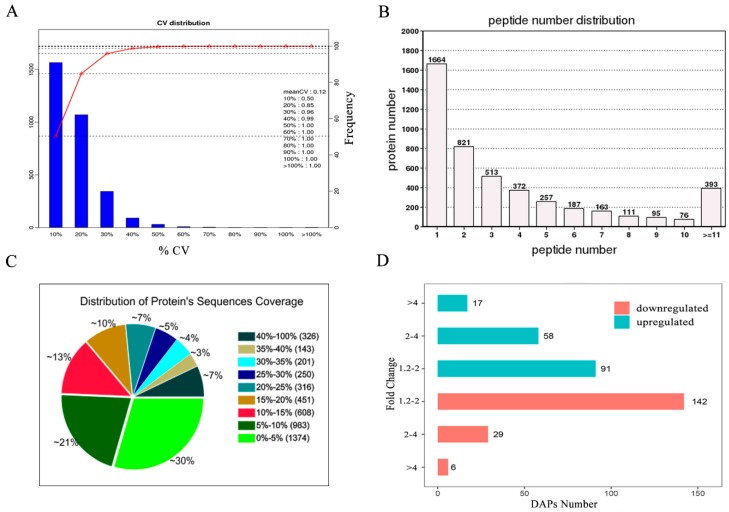
(**A**) The repeatability of the three protein abundance ratios (HBF/NBF). The x-axis represents % CV of the three ratios. The left vertical axis represents the number of proteins. The right vertical axis represents the cumulative % of the counted proteins (red line). (**B**) The peptide number distribution. (**C**) The distribution of protein’s sequences coverage. (**D**) The number of proteins identified up and down-regulated between the HBF and NBF placentas.

Using iTRAQ technology, we detected 4652 proteins in porcine placentas. The detailed information of the identified proteins is shown in Table S2. More than 85% of the identified proteins had a CV value <20% among the three independent comparisons (Figure 2A). The distribution of peptide number and protein’s sequences coverage are shown in Figure 2B,C, respectively. A total of 343 proteins were identified as DAPs (>1.2 fold change, *p* < 0.05), of which 166 were up-regulated and 177 were down-regulated between the HBF and NBF placentas (Figure 2D). The detailed results of the DAPs are displayed in Table S3.

### 3.4. GO and KEGG Analyses of the DAPs

**Figure 3:**
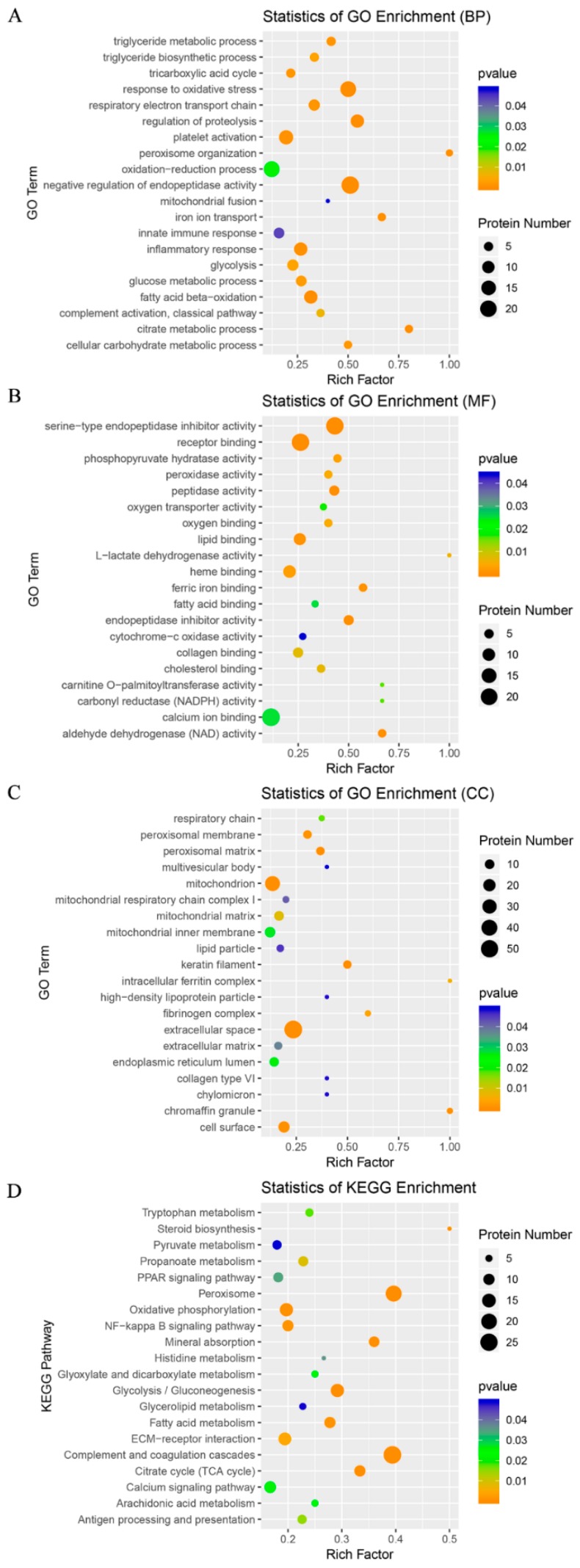
Gene Ontology (GO) and Kyoto Encyclopedia of Genes and Genomes (KEGG) enrichment analysis of the differentially abundant proteins (DAPs). The enrichment analysis of the DAPs was conducted using a hypergeometric test on the basis of the annotated results from the GO and KEGG databases, respectively. (**A**) The most representative 20 GO terms under biological process (BP). (**B**) The most representative 20 GO terms under molecular function (MF). (**C**) The most representative 20 GO terms under cellular component (CC). (**D**) The most representative 20 KEGG pathways.

To obtain a more comprehensive understanding of the DAPs, we performed GO and KEGG enrichment analyses to identify potential pathways that could be affected by the DAPs. The 325 types of DAPs (94.8%) were significantly enriched in 204 GO terms (*p* < 0.05), which included 120 BP terms, 50 MF terms and 34 CC terms (Table S4). The 20 most representative and markedly enriched GO terms within the three main functional modules are shown in Figure 3A–C. The leading seven BP terms were ‘fatty acid beta-oxidation’, ‘tricarboxylic acid cycle’, ‘inflammatory response’, ‘glycolysis’, ‘respiratory electron transport chain’, ‘triglyceride biosynthetic process’ and ‘response to oxidative stress’ (Figure 3A). A total of 245 DAPs (71.4%) were associated with 179 specific KEGG pathways (Table S5). The attractive pathways included ‘oxidative phosphorylation’, ‘glycolysis/gluconeogenesis’, ‘peroxisome’ and ‘steroid biosynthesis’ (Figure 3D). These results demonstrated that a large portion of the DAPs were associated with energy and substance metabolism, stress response and innate immunity, and they were mainly located in the mitochondria and extracellular space.

### 3.5. Protein Interaction Networks of the DAPs

**Figure 4:**
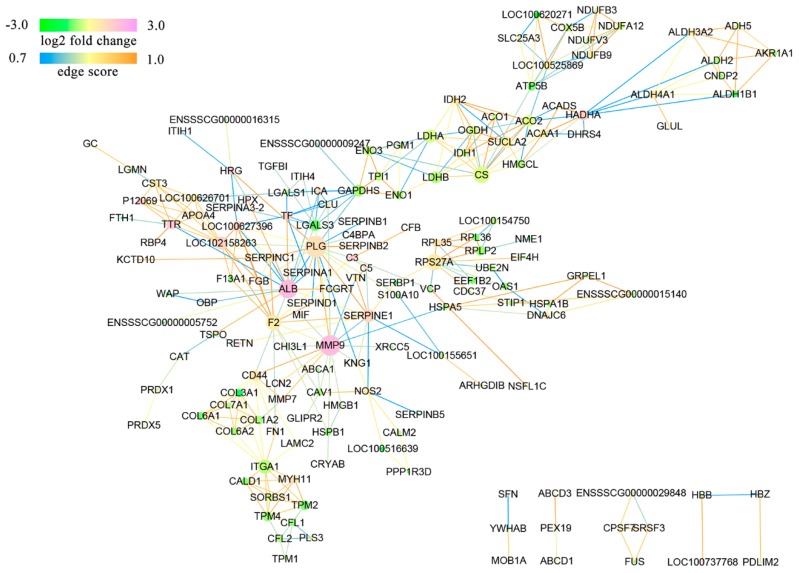
The protein–protein interaction networks of the DAPs were built using STRING 10.5 with a combined score larger than 0.7 (high confidence) and visualized with Cytoscape 3.4.0. DAPs are represented by round nodes. The pink node indicates up-regulation and the green node indicates down-regulation of the DAPs. The node size indicates high interaction degree (large) or low interaction degree (small). Proteins are connected with each other by an edge, which color indicates the combined score.

As shown in Figure 4, the networks obtained without unconnected proteins or self-loops included 159 nodes and 331 edges. Nodes with a high degree of interaction may play an important role in the regulation of a network. Using PPI networks analysis, we obtained many central nodes, including matrix metalloproteinase-9 (MMP9), lactate dehydrogenase (LDHB) and citrate synthase (CS).

### 3.6. Validation of the DAPs Using RT-qPCR and ELISA Analysis

**Figure 5:**
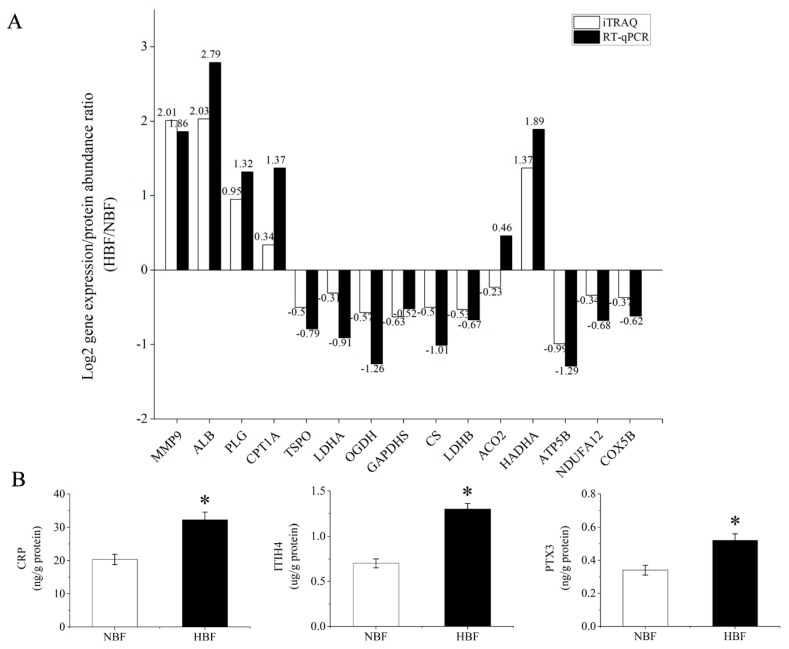
Validation of the DAPs using RT-qPCR and ELISA analysis. (**A**) Fifteen DAPs between the HBF and NBF placentas were selected. The expression ratios (HBF/NBF) of the genes encoding these DAPs are shown in parallel with protein abundance ratios identified by iTRAQ proteomics for comparison convenience. Log2 gene expression/protein abundance ratio (HBF/NBF) >0 indicates that the gene expression/protein abundance is up-regulated in the HBF placentas compared to the NBF placentas. (**B**) The expression levels of CRP, ITIH4 and PTX3 obtained at ELISA analysis. n = 30 biological replicates in each group. * *p* < 0.05.

To further validate whether the protein abundance differentiation determined by iTRAQ was consistent with the transcript level of the corresponding gene, we performed a transcriptional analysis of 15 selected DAPs using RT-qPCR. We chose these proteins because of their high interaction degree in PPI networks and their potentially important functions. As shown in Figure 5A, 14 genes showed the same trend between their transcriptional levels and the abundance of the corresponding proteins. In addition, we measured the expression of three acute-phase proteins (CRP, ITIH4, PTX3) in the HBF and NBF placentas by ELISA. The expression levels of three acute-phase proteins obtained upon ELISA analysis confirmed the trend obtained by iTRAQ (Figure 5B). The RT-qPCR and ELISA results partially validated the iTRAQ data and indicated that our proteomic findings were reliable.

## 4. Discussion

Studies have shown that the incidence of maternal obesity is increasing in human beings [1], and an abnormal intrauterine environment induced by maternal obesity may lead to placental dysfunction [22], which is associated with poor short- and long-term outcomes of both mothers and infants. Similarly to the research in humans, studies have suggested that in pigs, there is a significant negative correlation between a sow’s backfat and subsequent reproductive performance [3]. In this study, the fewer number of live-born piglets observed in the HBF Yorkshire sows corroborated previous results obtained from Landrace sows [13]. We also found that HBF sows had an increased percentage of stillborn piglets, a higher number of LBW piglets, a greater within-litter variation of piglet weights compared to the NBF sows. Based on previous studies [6] and our results, we reasonably deduced that the higher percentage of stillborn piglets may have been due to the birth canal structures and weak uterine contractions in the obese sows and greater within-litter variation of piglet weights. Collectively, these findings strongly demonstrate that exorbitant fat accumulation at mating in pigs is not conducive to the improvement of reproductive performance. It has been demonstrated that maternal lipid profiles are associated with fetal growth and development [23]. The higher maternal plasma TG and FFA contents observed in the obese sows in our trial are in agreement with previous studies in which high pre-pregnancy body mass index (BMI) [24] and pregnancy complicated by intrauterine growth restriction [25] increased the maternal plasma levels of TG and FFA. In addition, we found higher maternal proinflammatory mediators, including IL-6 and TNF-α, in obese sows, in agreement with a previous report in which increasing maternal BMI was associated with systemic inflammation [26]. In this study, placental efficiency was decreased in the obese sows, which indicates the presence of placental abnormality. Inappropriate maternal metabolic hormones and adipokines are associated with abnormal placental function.

Considering that the placenta can mediate the effects of maternal obesity on a fetus, unveiling the molecular mechanisms underlying placental abnormality is of great significance to clinical obstetrics. Although the effects of maternal obesity on the placental proteome in humans have been investigated previously by traditional two-dimensional electrophoresis (2D-DIGE) [19], the iTRAQ protein quantitative technique is more accurate than low-throughput proteomic methods, and can provide higher resolution results. In this study, using iTRAQ coupled with 2DLC-MS/MS, we compared the quantitative changes in the proteome of the placenta in response to obese pregnancy in sows. Globally, we identified 343 DAPs between the HBF and NBF placentas, which were predicted using bioinformatics to change many vital biological processes. Given the great quantity of biological processes predicted to be disturbed in this study, we focused on five types of DAPs that are involved in nutrient metabolism, mitochondrial function, steroid hormone biosynthesis, oxidative stress and inflammation, respectively. These proteins and potential pathways are discussed below.

### 4.1. Carbohydrate and Lipid Metabolism-Related DAPs

The placenta, especially the syncytiotrophoblast within it, has high metabolic activity, the energy for which is derived from aerobic respiration in the mitochondria. Although glucose is the major substrate for energy generation, the placenta can also generate energy via fatty acid oxidation (FAO). In the present study, a group of DAPs was involved in carbohydrate and lipid metabolism, including glycolysis, tricarboxylic acid (TCA) cycle, fatty acid metabolism and TG biosynthetic process. Our proteomic results showed that a total of eight catalytic enzymes (Table 2) which participated in the glycolysis were down-regulated in HBF placentas. The down-regulation of these eight proteins may indicate a decreased rate of glycolysis. It is well-known that the tricarboxylic acid (TCA) cycle is a central pathway in energy metabolism. In this study, most of the DAPs related to the TCA cycle were down-regulated in HBF placentas, including citrate synthase (CS) and oxoglutarate dehydrogenase (OGDH) (Table 2). Among these proteins, CS and OGDH, the key enzymes in TCA, play a primary role in maintaining energy production. A reduced abundance of CS leads to excessive superoxide formation and cell apoptosis [27]. To the best of our knowledge, we were the first to find that several key enzymes involved in glycolysis and the TCA cycle are down-regulated in the placentas of obese sows. Whether dysregulation of these catalytic enzymes results in placental dysfunction and adverse outcomes for these fetuses needs to be further verified in future studies.

Previous studies have indicated that obese pregnancy contributes to placental lipotoxicity [8]. Similarly, in this study, an increased TG content was observed in the HBF placentas. Furthermore, five DAPs (Table 2) involved in the TG biosynthetic process, were up-regulated in the HBF placentas, including key lipogenic enzymes fatty acid synthase (FASN) and ATP-citrate synthase (ACLY). FASN, as a key lipogenic enzyme, catalyzes the terminal steps in the de novo synthesis of fatty acids using acetyl-CoA as a primer. ACLY is responsible for the synthesis of acetyl-CoA and has a central role in de novo lipid biosynthesis. These results demonstrated that HBF placentas have significantly increased fatty acid synthesis and TG ectopic deposition. Concurrently, 10 DAPs (Table 2) in the peroxisome involved in FAO were up-regulated, which may indicate greater peroxisomal FAO in placentas of obese sows. This result was in agreement with a previous report in which peroxisomal FAO is greater in placentas of obese women [28]. Three of the four DAPs (Table 2) involved in mitochondrial FAO were up-regulated, including Carnitine palmitoyltransferase I (CPT1A), which is a rate-limiting catalyticase of mitochondrial long-chain fatty acids beta-oxidation. Higher CPT1A protein abundance seemed to suggest that mitochondrial FAO was also notably increased in HBF placentas. However, previous studies showed that the post-translational modifications of CPT1A caused by lipid peroxidation and malonyl-CoA could decrease CPT1A activity and reverse initially activated FAO [29,30]. According to these studies, we speculate that mitochondrial FAO was still not enhanced in HBF placentas and greater peroxisomal FAO may be a compensatory mechanism [28]. Furthermore, the byproduct of peroxisomal FAO is hydrogen peroxide, which can lead to further cellular damage. Increased placental lipid esterification induced by maternal obesity may limit the amount of maternal lipid transferred to the fetus. Collectively, our study showed that maternal lipid overload is associated with abnormal carbohydrate and lipid metabolism and mitochondrial dysfunction in the placenta.

### 4.2. Mitochondrial Respiratory Chain and Mitochondrial Morphology-Related DAPs 

The mitochondrial respiratory chain transfers electrons from NADH and succinate to molecular oxygen, coupling energy currency ATP production, which is essential for normal cells. Many studies have indicated that defective mitochondrial respiratory chains are associated with the pathogenesis of many diseases, such as nonalcoholic steatohepatitis. In this study, the proteomic results showed that HBF placentas have decreased abundance of mitochondrial electron transport chain subunits, including complexes I, II, III, IV and ATP synthase (Table 2). In particular, the decreased abundance of ATP synthase (ATP5B), which synthesizes ATP in the presence of a transmembrane proton gradient in the placentas of obese sows, coincides with previous 2D-DIGE results in obese mothers [19]. Recent findings have indicated that the inflammatory intrauterine environment associated with maternal obesity leads to the inhibition of mitochondrial respiration in these placentas [31]. Additionally, FFA have been shown to affect mitochondrial respiration by increasing the production of reactive oxygen species (ROS) [32] and mitochondrial proton conductance (uncoupling) [33]. Thus, it is not surprising that dysregulation of several proteins involved in the respiratory chain has been observed in the HBF placentas. It is well-known that a significant reduction of electron carriers will result in an overproduction of superoxide. This coincides with our and other research in which overproduction of free radicals was observed in obese placentas. We speculate that a reduced abundance of placental mitochondrial respiratory chain subunits compromises placental function and affects the developmental programming of the fetus. Growing evidence links mitochondrial dynamics to bioenergetic adaptation, and mitochondrial morphology depends on the balance of mitochondrial fusion and fission. Interestingly, the abundance of DAPs involved in mitochondrial fusion were increased in HBF placentas (Table 2), which differs from a previous study of brain tissue [34] in which excess nutrient environments contributed to the fragmentation of mitochondria. We speculate that this condition may be an adaptive response to respiratory chain inhibition or tissue specificity.

### 4.3. Steroid Hormone Biosynthesis-Related DAPs

Steroid hormones, especially estradiol and progesterone which are synthesized in the placenta during pregnancy, play an extremely critical role in pregnancy maintenance. They can suppress the maternal immune system against the fetus and promote the quiescence of the myometrium. Previous studies have indicated that plasma concentrations of both progesterone and estradiol are decreased in obese pregnant women [35]. In line with that report, we observed that plasma estradiol and progesterone concentrations were significantly lower in obese sows (unpublished data). In this study, our proteomic results showed that several DAPs involved in steroid hormone biosynthesis and regulation were down-regulated in the HBF placentas, including STAR-related lipid transfer protein (STAR) and mitochondrial translocator protein (TSPO) (Table 2). As we all know, the rate-limiting step in the biosynthesis of steroid hormones is the transfer of cholesterol from the cytoplasm into the mitochondria. STAR performs the function of a cholesterol transporter, importing cholesterol, the primary biosynthetic precursor for oestrogen and progesterone, into the mitochondria. TSPO, as a steroid biosynthesis regulator, facilitates cholesterol transport. Thus, these proteins may play an important role in the regulation of decreased steroid hormone biosynthesis in obese placentas.

### 4.4. Oxidative Stress-Related DAPs

Our results indicated that maternal obesity could result in impaired antioxidant ability and oxidative stress in porcine placenta, as evidenced by the higher MDA and lower T-AOC in the HBF placentas. In this study, 18 types of DAPs involved in oxidative stress were observed between the HBF and NBF placentas (Table 2). The iron-binding protein, ferritin, helps to prevent the formation of ROS by maintaining labile iron (Fe^2+^), which is harmful because of its propensity to generate highly damaging free radicals in a soluble and unreactive Fe^3+^ form. In a previous study, down-regulation of ferritin was observed in obese placentas [19], which might explain aberrant iron content in the fetuses of obese women [36]. Copper transport protein (ATOX1) is a significant component of cellular antioxidant defense [37]. Our proteomic results showed a decrease in the abundance of ferritin (FTL and FTH1) and ATOX1, which may enhance the generation of ROS in obese placentas. The DJ-1 protein plays an important role in cell protection against oxidative stress and mitochondrial stresses [38]. Heat shock protein B1 (HSPB1), an ATP-independent chaperone, can maintain certain denatured proteins in a folding-competent state and enhance the thermostability of a cell [39]. Heat shock protein A8 (HSPA8), a molecular chaperone, participates in the folding and transport of newly synthesized polypeptides and chaperone-mediated autophagy. Heat shock protein A5 (HSPA5) plays an important role in facilitating the assembly of multimeric protein complexes inside the endoplasmic reticulum [40]. In this study, the above-mentioned four proteins involved in defending cells against oxidative stress were down-regulated in obese placentas, which coincides with a defective antioxidant capacity. Along with these findings, the activities of CAT and SOD were reduced compared to the NBF placentas. Interestingly, the abundance and activity of GSH-PX were significantly increased in the placentas of the obese sows, which may have been a compensatory response. Consequently, our results indicated that maternal obesity could result in increased placental oxidative stress, because of impaired antioxidant ability and the overproduction of free radicals.

### 4.5. Inflammatory Response-Related DAPs

In nonpregnant individuals, obesity is considered a chronic, low-grade inflammatory state. A physiological inflammatory state becomes enhanced in pregnancy complicated by obesity. Recent studies have demonstrated that maternal obesity enhances proinflammatory cytokines contents in maternal circulation and the placenta [26]. Consistent with previous studies, our results showed that HBF sows had significantly increased IL-6 and TNF-α levels in the blood and placental tissues. Proteomic analysis revealed that a total of 12 DAPs were involved in the inflammatory response (Table 2). Azurocidin (AZU1), also known as HBP/CAP37, can cause an increase in microvascular permeability and attract monocytes to inflammation sites when released. Complement anaphylatoxin C5a can induce inflammatory reactions via the induction of histamine release and the production of pro-inflammatory mediators [41]. Kininogen 1 (KNG1) plays a significant role in the pathogenesis of chronic inflammation involved in cytokine secretion, cellular injury and the release of proteases. In this study, the proteomic analysis revealed a significant increase of AZU1, C5a and KNG1 in HBF placentas. Proteomic results showed that a group of acute-phase proteins involved in inflammatory responses were up-regulated in the HBF placentas including C-reactive protein (CRP), major acute-phase protein (ITIH4), Pentraxin-3 (PTX-3) and Alpha-1-antitrypsin (SERPINA1). CRP, a sensitive biomarker of inflammation and tissue damage, has anti-inflammatory effects [42]. Its biosynthesis is up-regulated under inflammatory mediators originating at the site of pathology. ITIH4, which has acted as an anti-inflammatory cytokine in some studies [43], plays a part in protecting uterus tissue from inflammatory injury during early pregnancy in pigs [44]. PTX-3, typically released at original inflammatory sites by several cell types and induced by both IL-l and TNF-α, plays a significant role in the clearance of apoptotic cells, inflammation and pregnancy. SERPINA1, an acute-phase glycoprotein, can inhibit neutrophil elastase [45] and suppress TNF-α production in endothelial lung cells [46]. The increased abundance of these four acute-phase proteins in the present study indirectly demonstrated that maternal obesity strengthened the inflammatory response in porcine placentas. Collectively, the increased pro-inflammatory mediators and decreased anti-inflammatory molecules in the current study suggested an increase in inflammation in HBF placentas. Maternal obesity induces inflammation, producing various pro-inflammatory factors that aggravate placental damage. Therefore, efforts which can reduce the damage in response to inflammation are important for the improvement of placental function. 

As discussed earlier, maternal obesity disturbs a large number of proteins/potential biological processes that may disrupt normal placental function, thus impacting fetal development. Using PPI networks analysis, we obtained many central nodes, including MMP9, LDHB and CS. MMP9 is important for embryo implantation [47], leukocyte migration and degranulation, regulating the release of cytochrome c from mitochondria and the cellular response to ROS [48]. LDHB and CS are vital catalytic enzymes which participate in glycolysis and the TCA cycle. These DAPs participate in many biological processes and may play important roles in the regulation of obesity-induced placental dysfunction.

## 5. Conclusions

Maternal obesity in pigs is not conducive to the improvement of reproductive performance and is associated with impaired maternal metabolism and decreased placental efficiency. Furthermore, exposure to an obese environment in utero is related to proteome alterations in the porcine placenta. We identified 343 deranged proteins using proteomics, which were predicted using bioinformatics to change many vital biological processes, including abnormal carbohydrate and lipid metabolism, mitochondrial dysfunction, decreased steroid hormone biosynthesis, and increased oxidative stress and inflammation.

## Figures and Tables

**Table 1:** Characteristics of the studied sows.

**Reproductive performance parameters**	**NBF**	**HBF**
Backfat at mating, mm	18 ± 0.4	25 ± 0.5 ^#^
Backfat at farrowing, mm	20 ± 0.5	27 ± 0.6 ^#^
Backfat gain during pregnancy, mm	1.54 ± 0.32	1.61 ± 0.31
Total born piglets, n	423	421
Live-born piglets, n	371	362
Litter size of live-born and stillborn piglets, n	14.12 ± 0.47	14.04 ± 0.39
Litter size of live-born piglets, n	12.37 ± 0.31	12.08 ± 0.32 *
Litter weight of live-born piglets, kg	17.15 ± 0.35	16.99 ± 0.64
Litter weight of live-born and stillborn piglets, kg	18.94 ± 0.51	18.78 ± 0.53
Average weight of live-born piglets, kg	1.51 ± 0.10	1.39 ± 0.09 *
Piglets with weights < 0.9 kg, n	0.96 ± 0.08	1.31 ± 0.07 ^#^
Rate of piglets with weight < 0.9 kg, % ^1^	7.17 ± 0.87	10.32 ± 0.93 *
CV for weights of live-born piglets, % ^2^	20.06 ± 1.69	23.38 ± 1.63 *
Placental weight, g	286.29 ± 4.07	346.97 ± 5.66 *
Placental efficiency ^3^	4.96 ± 0.19	4.39 ± 0.24 ^#^
**Maternal plasma parameters**	**NBF**	**HBF**
Glucose, mmol/L	4.29 ± 0.23	4.35 ± 0.22
HDL, mM	1.27 ± 0.10	1.25 ± 0.08
LDL, mM	4.17 ± 0.27	4.20 ± 0.21
CHOL, mM	5.97 ± 0.54	6.11 ± 0.51
TG, mM	0.78 ± 0.15	0.89 ± 0.13 *
FFA, mM	0.14 ± 0.07	0.22 ± 0.04 *
IL-6, ng/L	237.69 ± 9.18	267.37 ± 9.29 *
TNF-α, ng/L	64.87 ± 3.23	97.19 ± 4.04 ^#^

NBF: normal backfat thickness at mating; HBF: high backfat thickness at mating; Values are mean ± SD, (* *p* < 0.05 vs. NBF, ^#^ *p* < 0.01 vs. NBF). ^1^ For analyses, the square root arcsine transformation for proportions was used. ^2^ The coefficient of variation (CV) for the piglets’ weights was calculated by dividing the standard deviation (SD) of each piglet’s weight within the litter by the average of those values, expressing it as a percentage. ^3^ A ratio between the birth weight (g) and the placental weight (g).

**Table 2:** DAPs involved in carbohydrate and lipid metabolism, mitochondrial function, steroid hormone biosynthesis, oxidative stress and inflammation.

NO.	Uniprot Accession	Score	Description	Gene	Ratio Average ^1^	*p*-Value
DAPs involved in glycolysis						
1	F1S814	699	Phosphoglucomutase-1	*PGM1*	0.73	0.001
2	D0G7F6	1557	Triosephosphate isomerase	*TPI1*	0.73	0.001
3	F1RM74	281	Glyceraldehyde-3-phosphate dehydrogenase	*GAPDHS*	0.65	<0.001
4	I3LK59	4047	Enolase	*ENO1*	0.65	0.001
5	Q1KYT0	1980	Beta-enolase	*ENO3*	0.73	0.001
6	P00336	1026	l-lactate dehydrogenase B chain	*LDHB*	0.69	<0.001
7	F1RII7	21595	Hemoglobin subunit beta	*HBB*	0.65	0.001
8	P00339	1468	l-lactate dehydrogenase A chain	*LDHA*	0.81	<0.001
DAPs involved in tricarboxylic acid cycle						
1	P00889	929	Citrate synthase, mitochondrial	*CS*	0.71	<0.001
2	I3LJW4	2855	Aconitase 1	*ACO1*	0.70	<0.001
3	F1SRC5	1602	Aconitate hydratase, mitochondrial	*ACO2*	0.63	0.004
4	Q0QF01	784	Succinate dehydrogenase flavoprotein subunit	*SDHA*	0.76	0.001
5	F1SSH8	1077	Oxoglutarate dehydrogenase	*OGDH*	0.67	<0.001
DAPs involved in triglyceride biosynthetic process						
1	A5YV76	6249	Fatty acid synthase	*FASN*	1.49	<0.001
2	G1FJ20	4204	ATP-citrate synthase	*ACLY*	1.48	<0.001
3	C0KEG5	98	1-acylglycerol-3-phosphate *O*-acyltransferase 7	*LPCAT4*	1.51	0.001
4	I3LLU0	495	Glycerol-3-phosphate dehydrogenase	*GPD1L*	1.57	<0.001
5	F1RWN0	159	Uncharacterized protein	*AGPAT2*	1.32	<0.001
DAPs involved in fatty acid oxidation						
1	A7J0B0	1777	Acyl-coenzyme A oxidase	*ACOX1*	1.88	0.002
2	Q28956	1346	17beta-estradiol dehydrogenase	*HSD17B4*	1.94	<0.001
3	D0G7F1	981	Sterol carrier protein 2	*SCP2*	1.49	0.001
4	F1RRB7	1262	Acetyl-CoA acyltransferase 1	*ACAA1*	1.48	<0.001
5	F1S3F7	173	ATP-binding cassette sub-family D member 4	*ABCD4*	1.69	<0.001
6	I3LI20	242	ATP-binding cassette sub-family D member 3	*ABCD3*	1.58	0.001
7	F1S2A1	71	ATP binding cassette subfamily D member 1	*ABCD1*	1.63	<0.001
8	F1SQH7	287	Solute carrier family 27 member 2	*SLC27A2*	1.57	0.005
9	Q3Y5G5	447	Peroxisomal enoyl coenzyme A hydratase 1	*ECH1*	1.45	0.012
10	F1SBY8	157	Peroxisomal carnitine *O*-octanoyltransferase	*CROT*	1.42	0.011
11	F1RJH2	366	Short-chain-specific acyl-CoA dehydrogenase	*ACADS*	1.53	0.002
12	Q29554	3820	Trifunctional enzyme subunit alpha, mitochondrial	*HADHA*	2.59	0.001
13	Q95JG9	481	Carnitine palmitoyltransferase I	*CPT1A*	1.27	<0.001
14	B2ZF49	3810	Hydroxyacyl-coenzyme A dehydrogenase	*HADH*	0.16	<0.001
DAPs involved in mitochondrial respiratory chain and mitochondrial morphology						
1	G8IFA6	228	Mitochondrial NADH dehydrogenase Fe-S protein 4	*NDUFS4*	0.55	<0.001
2	I3LRR4	389	NADH dehydrogenase [ubiquinone] flavoprotein 3	*NDUFV3*	0.67	0.002
3	F1SQP4	264	NADH dehydrogenase 1 alpha subcomplex subunit 12	*NDUFA12*	0.66	0.001
4	Q0QF01	784	Succinate dehydrogenase flavoprotein subunit	*SDHA*	0.76	0.001
5	F1RPD4	340	Cytochrome b-c1 complex subunit 2, mitochondrial	*UQCRC2*	0.17	<0.001
6	Q5S3G4	309	Cytochrome c oxidase subunit 5B, mitochondrial	*COX5B*	0.55	0.001
7	F1SLA0	9014	ATP synthase subunit beta	*ATP5B*	0.50	0.001
8	F1RF76	54	Mitofusin 2	*MFN2*	2.08	0.003
9	F1SFG7	148	mitochondrial dynamin like GTPase	*OPA1*	1.28	<0.001
DAPs involved in steroid hormone biosynthesis and regulation						
1	F1SJR8	59	Translocator protein	*TSPO*	0.71	<0.001
2	F1RID6	57	StAR related lipid transfer domain containing 5	*STARD5*	0.60	<0.001
3	F1RSR5	28	StAR related lipid transfer domain containing 13	*STARD13*	0.68	0.003
DAPs involved in oxidative stress						
1	P79335	147	Plasminogen activator inhibitor 1	*SERPINE1*	2.30	0.005
2	F1SGS9	1461	Catalase	*CAT*	0.75	<0.001
3	I3LDC7	2004	Isocitrate dehydrogenase [NADP]	*IDH1*	0.73	<0.001
4	F1S131	165	Caspase 6	*CASP6*	0.51	0.006
5	Q0R678	860	DJ-1 protein	*PARK7*	0.59	0.001
6	F1SJT7	182	Apolipoprotein A-IV	*APOA4*	1.72	0.001
7	P67937	1612	Tropomyosin alpha-4 chain	*TPM4*	0.60	<0.001
8	F1SQP4	264	NADH dehydrogenase 1 alpha subcomplex subunit 12	*NDUFA12*	0.66	0.001
9	F2Z5B6	1159	Tropomyosin alpha-1 chain	*TPM1*	0.64	<0.001
10	Q6UJZ1	118	Glutathione peroxidase	*GPX3*	2.89	0.003
11	D0G0C7	172	Antioxidant protein 1 homolog	*ATOX1*	0.53	<0.001
12	F1RIP3	647	Ferritin	*FTL*	0.28	<0.001
13	I3LR69	68	Ferritin	*FTH1*	0.27	<0.001
14	F1S9Q3	3368	Heat shock cognate 71 kDa protein	*HSPA8*	0.64	<0.001
15	F1RS36	5142	78 kDa glucose-regulated protein	*HSPA5*	0.62	<0.001
16	F1SC56	147	Matrix metalloproteinase-9	*MMP9*	4.02	<0.001
17	Q5S1U1	1057	heat shock protein beta-1	*HSPB1*	0.55	<0.001
18	F1RKG8	781	Uncharacterized protein	*PEBP1*	0.65	<0.001
DAPs involved in inflammatory response						
1	F1SME1	122	Complement C5a anaphylatoxin	*C5*	2.21	<0.001
2	P79263	1833	Inter-alpha-trypsin inhibitor heavy chain H4	*ITIH4*	2.62	0.001
3	F1S131	165	Caspase 6	*CASP6*	0.51	0.006
4	A5D9N3	199	Allograft inflammatory factor 1	*AIF1*	0.59	0.001
5	F1SCF0	2964	Alpha-1-antitrypsin	*SERPINA1*	2.67	<0.001
6	A0MWC5	147	Monocyte differentiation antigen CD14	*CD14*	0.71	<0.001
7	F1RJ76	50	C-reactive protein	*CRP*	2.36	0.043
8	F1SJM0	315	Pentraxin-3	*PTX3*	3.53	0.018
9	F1SFI4	235	Kininogen 1	*KNG1*	2.01	0.003
10	P80015	429	Azurocidin	*AZU1*	5.18	<0.001
11	I3LL32	73	Uncharacterized protein	*CHIA*	1.75	<0.001
12	F1RUN2	7337	Serum albumin	*ALB*	4.08	<0.001

^1^ The average of the protein abundance ratios (HBF/NBF).

## Supplementary Materials

The following are available online at https://www.mdpi.com/2076-2615/9/8/546/s1, Figure S1: Position for backfat thickness measurement in live pigs (P2; 4–6 cm away from body midline at the last rib level). Figure S2: Flowchart of proteomic experimental design; each pooled biological replicate consists of five male placentas and five female placentas which were randomly selected from ten sows from the same backfat group; the pooled biological samples were labelled with iTRAQ reagents 114, 116, 118, 117, 119 and 121 respectively. Table S1: RT-qPCR primers. Table S2: List of 4652 proteins identified using iTRAQ. Table S3: List of 343 DAPs between the HBF and NBF placentas. Table S4: GO analysis of the DAPs. Table S5: KEGG pathway analysis of the DAPs.
