# Supplementary material for: The Effects of Maternal Obesity on Porcine Placental Efficiency and Proteome
Source: Animals (Basel). 2019 Aug 12;9(8):546. doi: 10.3390/ani9080546 (PMC6720507; doi:10.3390/ani9080546)
Supplement: Supplementary file 1 [file animals-09-00546-s001.zip › animals-542310 suppl/Graphical Abstract.pdf]

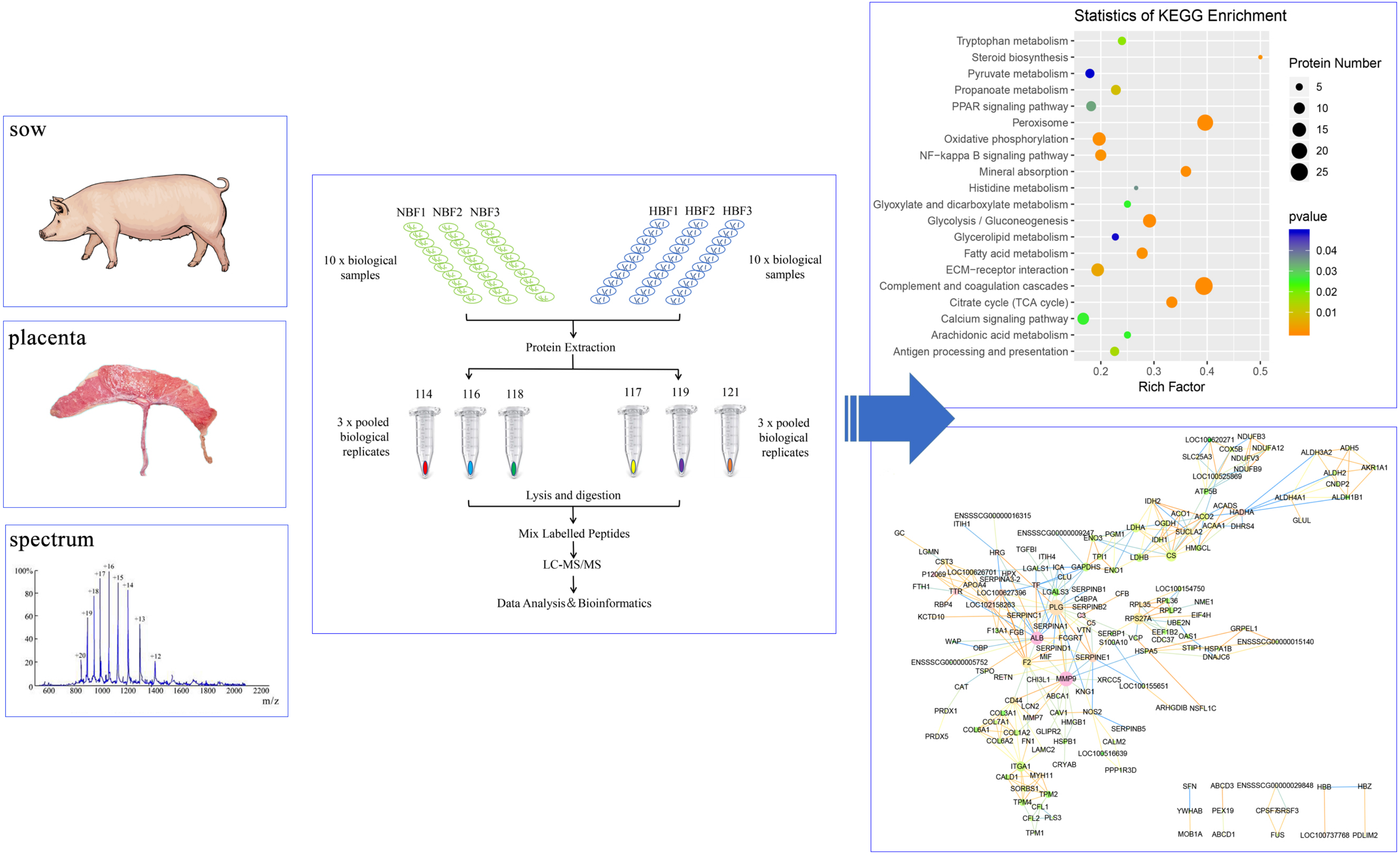
sow
placenta
spectrum
100%
80
60
40
20
0
600
800
1000
1200
1400
1600
1800
2000
2200
m/z
+20
+19
+18
+17
+16
+15
+14
+13
+12
NBF1 NBF2 NBF3
HBF1 HBF2 HBF3
10 x biological samples
10 x biological samples
Protein Extraction
114
116
118
117
119
121
3 x pooled biological replicates
3 x pooled biological replicates
Lysis and digestion
Mix Labelled Peptides
LC-MS/MS
Data Analysis & Bioinformatics
Statistics of KEGG Enrichment
Tryptophan metabolism
Steroid biosynthesis
Pyruvate metabolism
Propanoate metabolism
PPAR signaling pathway
Peroxisome
Oxidative phosphorylation
NF−kappa B signaling pathway
Mineral absorption
Histidine metabolism
Glyoxylate and dicarboxylate metabolism
Glycolysis / Gluconeogenesis
Glycerolipid metabolism
Fatty acid metabolism
ECM−receptor interaction
Complement and coagulation cascades
Citrate cycle (TCA cycle)
Calcium signaling pathway
Arachidonic acid metabolism
Antigen processing and presentation
0.2
0.3
0.4
0.5
Rich Factor
Protein Number
5
10
15
20
25
pvalue
0.04
0.03
0.02
0.01
LOC100620271 NDUFB3
COX5B NDUFA12
SLC25A3 NDUFV3
NDUFB9
LOC100525869
ATP5B
ALDH3A2 ADH5
ALDH2 AKR1A1
CNDP2
ALDH4A1 ALDH1B1
GLUL
IDH2
ACADS
ACO1 HADHA
ACO2
ACAA1 DHRS4
LDHA OGDH
SUCLA2
IDH1
HMGCL
LDHB CS
ENSSSCG00000016315
ITIH1
ENSSSCG00000009247
ENO3 PGM1
GC
LGMN
HRG TGFBI
ITIH4
TPI1
CST3
LGALS1 ICA GAPDHS ENO1
P12069 LOC100626701 HPX
CLU
SERPINA3-2
APOA4
TF
FTH1
TTR LOC100627396
LGALS3 SERPINB1
CFB
LOC100154750
C4BPA
RPL36 NME1
RBP4 LOC102158263
PLG SERPINB2
RPL35
RPLP2
EIF4H
KCTD10
SERPINC1
C3
RPS27A
UBE2N
C5
SERPINA1
GRPEL1
EEF1B2
F13A1 FGB
VTN
SERBP1
OAS1
ALB
FCGRT
VCP
CDC37
ENSSSCG00000015140
WAP
OBP
S100A10
STIP1 HSPA1B
SERPIND1
HSPA5
DNAJC6
MIF
SERPINE1
ENSSSCG00000005752
F2
TSPO
RETN
CHI3L1
MMP9
XRCC5
LOC100155651
CAT
KNG1
ABCA1
CD44
ARHGDIB NSFL1C
LCN2
PRDX1
COL3A1
CAV1
NOS2
MMP7
COL7A1
HMGB1
SERPINB5
COL6A1
COL1A2 GLIPR2
PRDX5
COL6A2
FN1
HSPB1
CALM2
LAMC2
LOC100516639
CRYAB
ITGA1
PPP1R3D
CALD1
MYH11
SFN
ABCD3 ENSSSCG00000029848 HBB
HBZ
SORBS1
TPM2
YWHAB
PEX19
CPSF7 SRSF3
TPM4
CFL1
CFL2 PLS3
MOB1A
ABCD1
FUS
LOC100737768 PDLIM2
TPM1
