# Supplementary figures and images for: The Effects of Maternal Obesity on Porcine Placental Efficiency and Proteome
Source: Animals (Basel). 2019 Aug 12;9(8):546. doi: 10.3390/ani9080546 (PMC6720507; doi:10.3390/ani9080546)

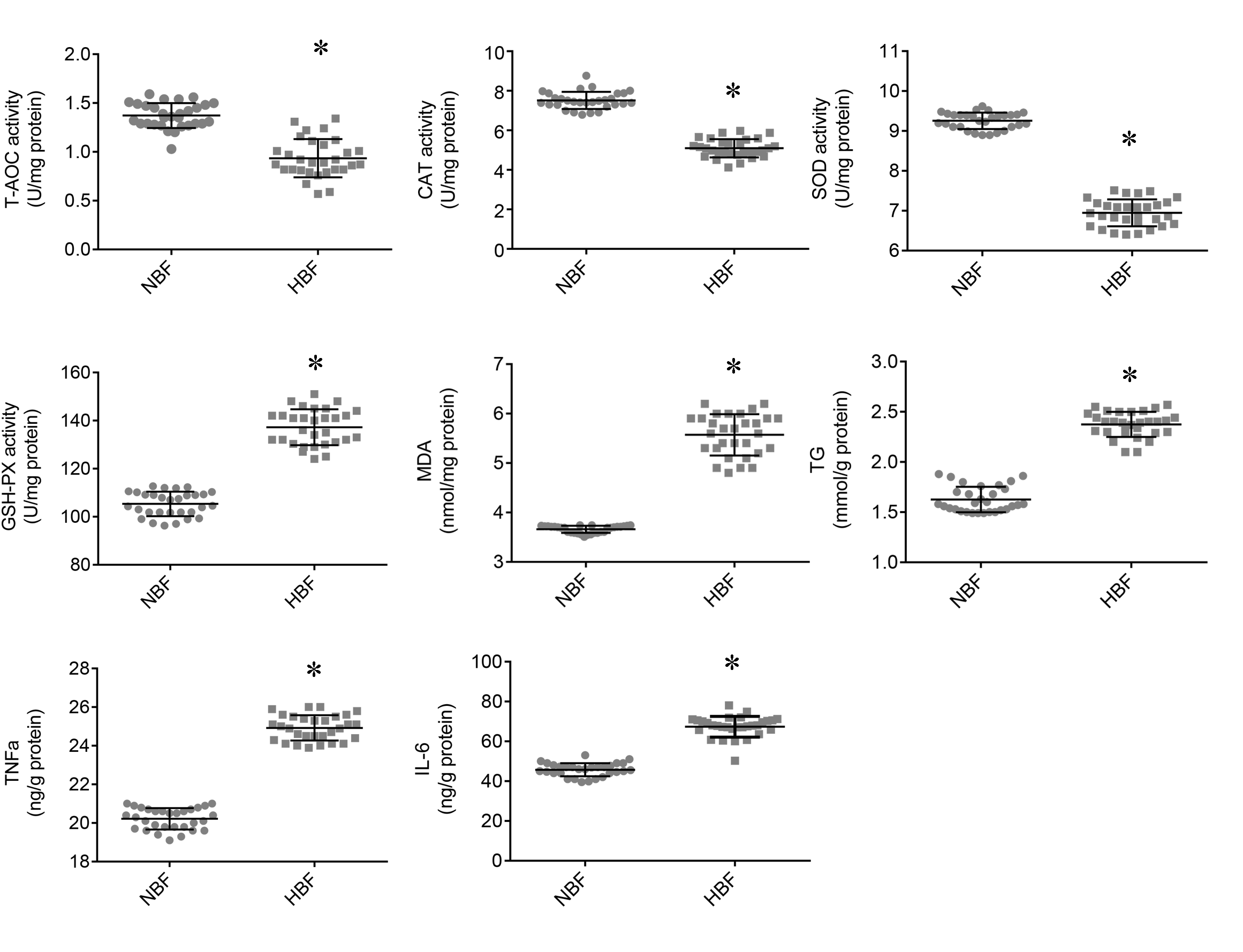

Supplement: Supplementary file 1 [file animals-09-00546-s001.zip › animals-542310 suppl/Figure 1.tif]

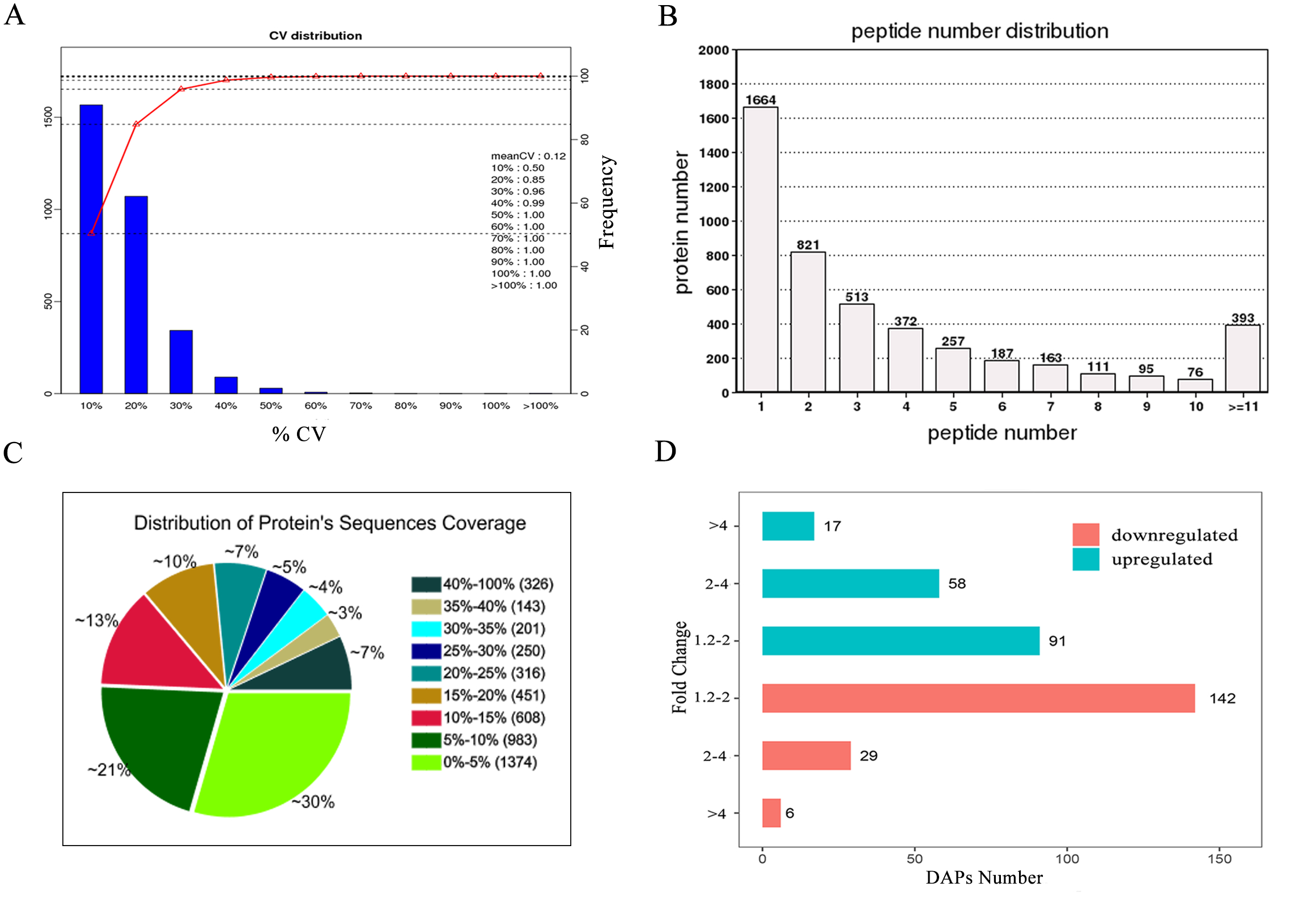

Supplement: Supplementary file 1 [file animals-09-00546-s001.zip › animals-542310 suppl/Figure 2.tif]

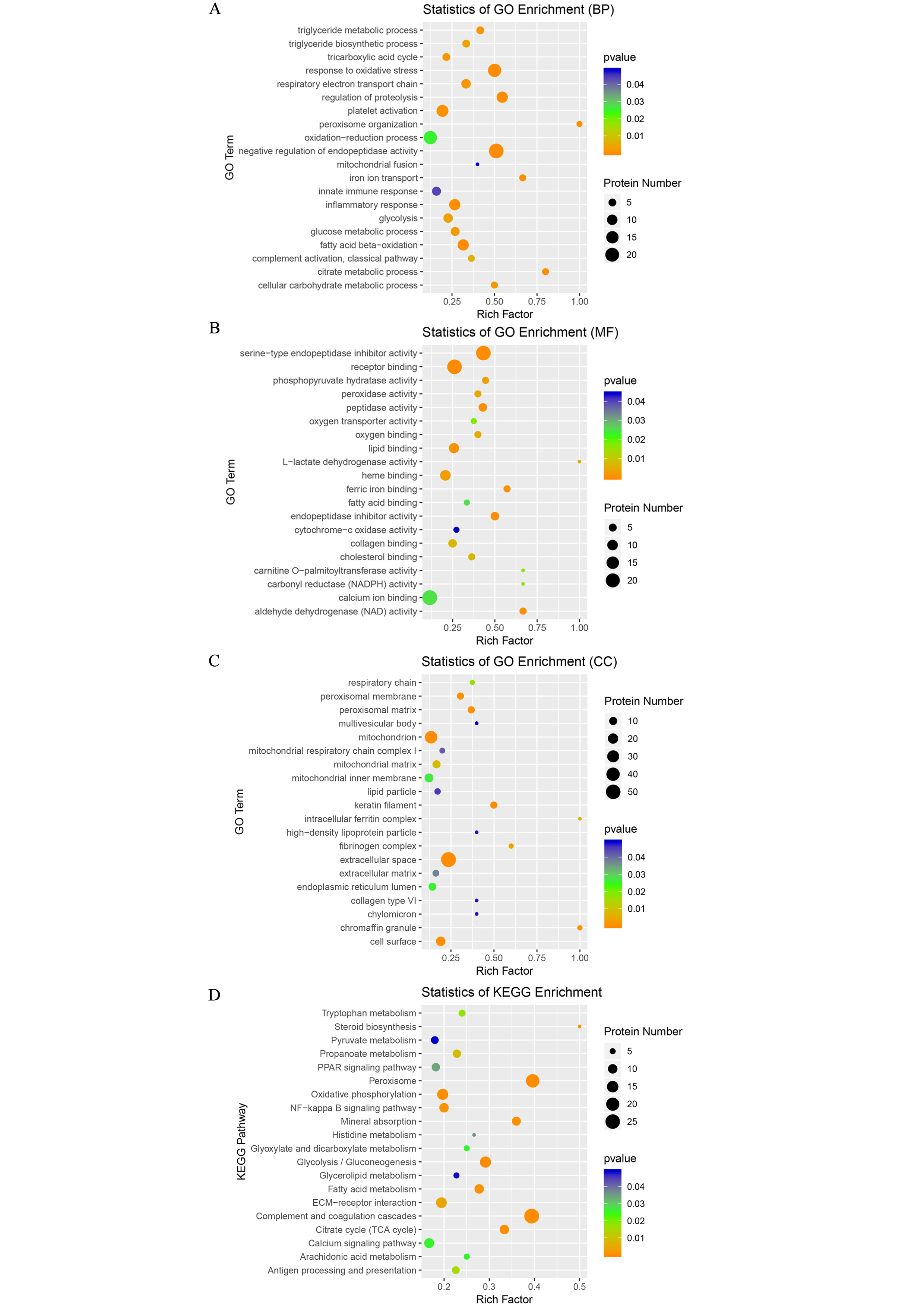

Supplement: Supplementary file 1 [file animals-09-00546-s001.zip › animals-542310 suppl/Figure 3.tif]

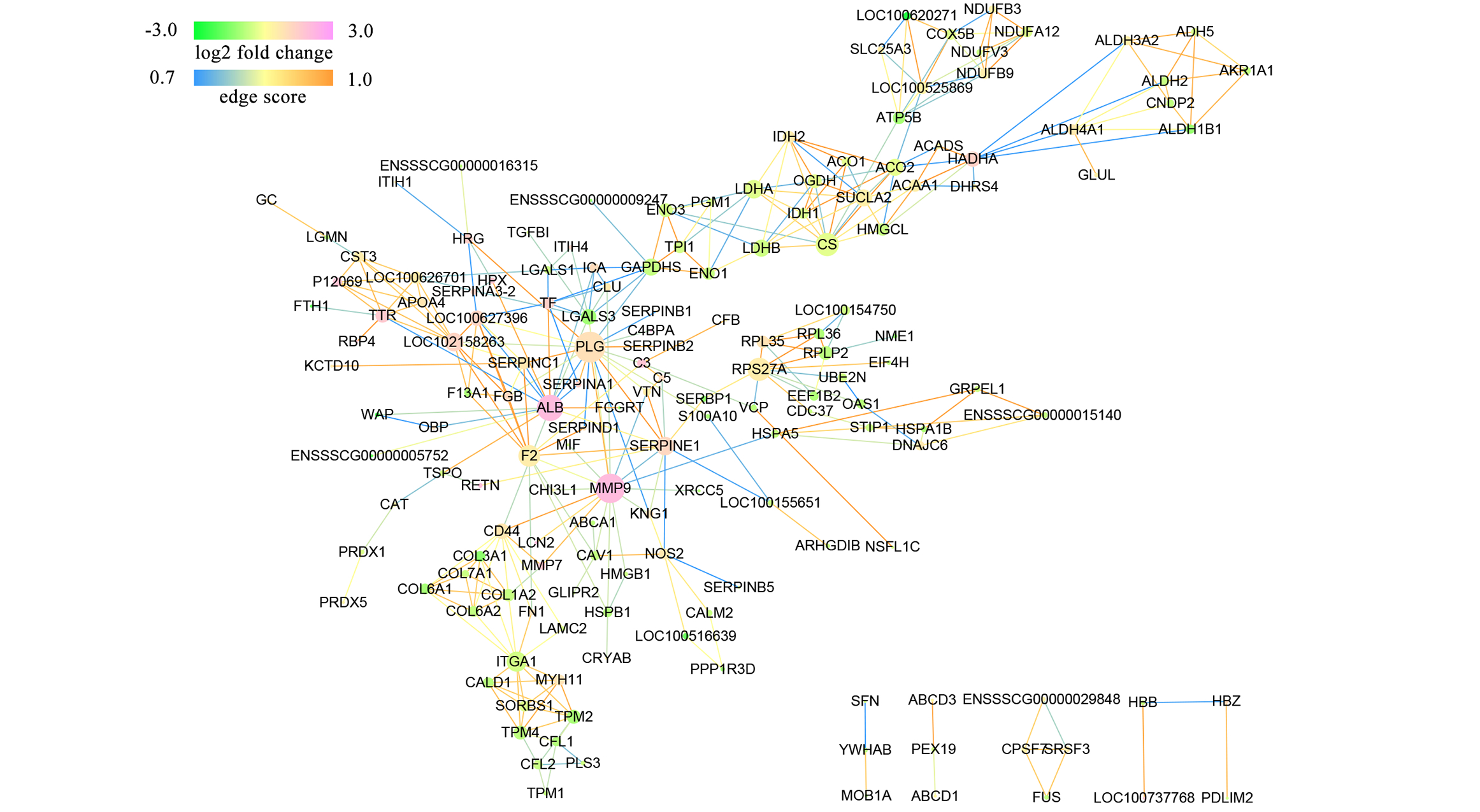

Supplement: Supplementary file 1 [file animals-09-00546-s001.zip › animals-542310 suppl/Figure 4.tif]

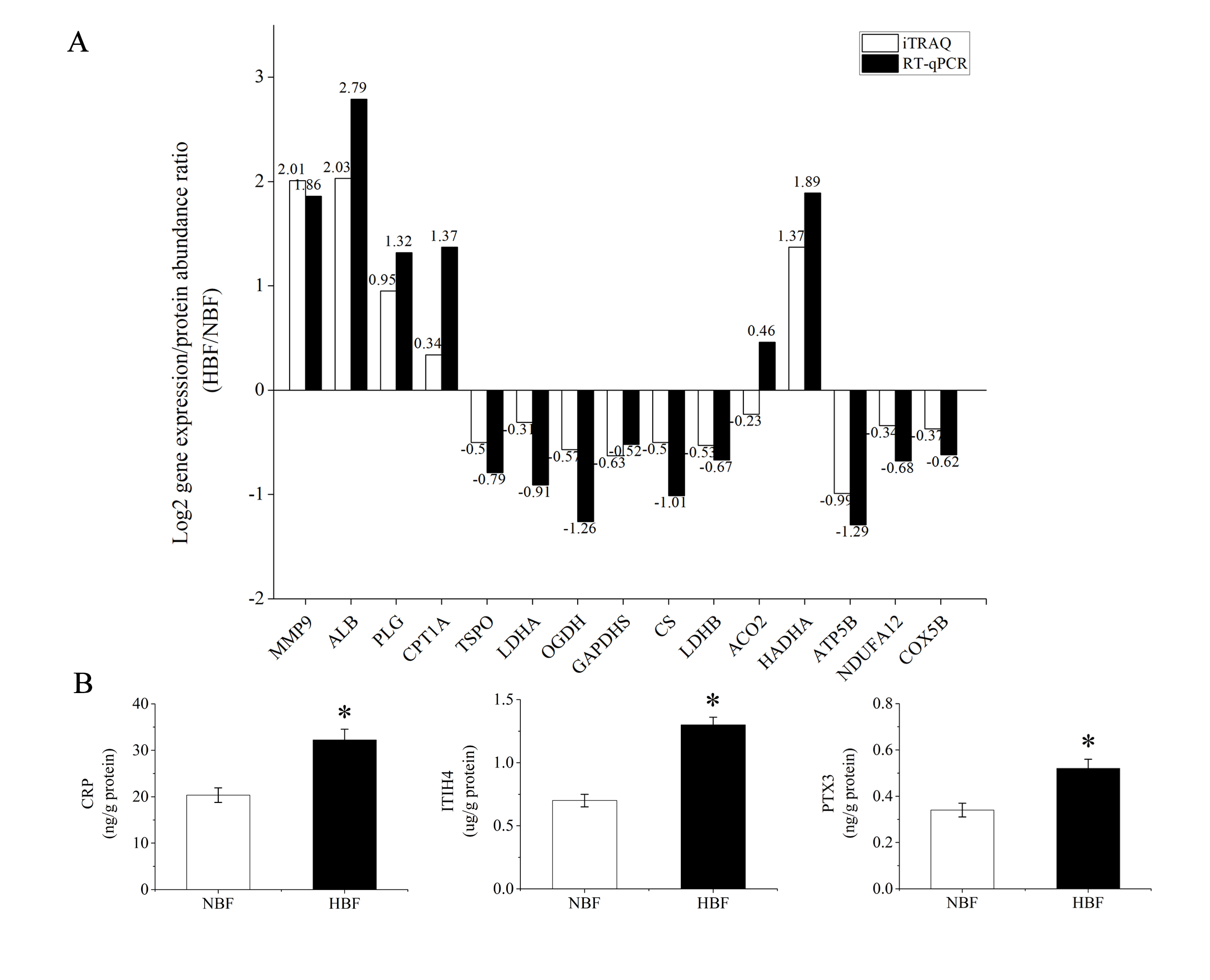

Supplement: Supplementary file 1 [file animals-09-00546-s001.zip › animals-542310 suppl/Figure 5.tif]

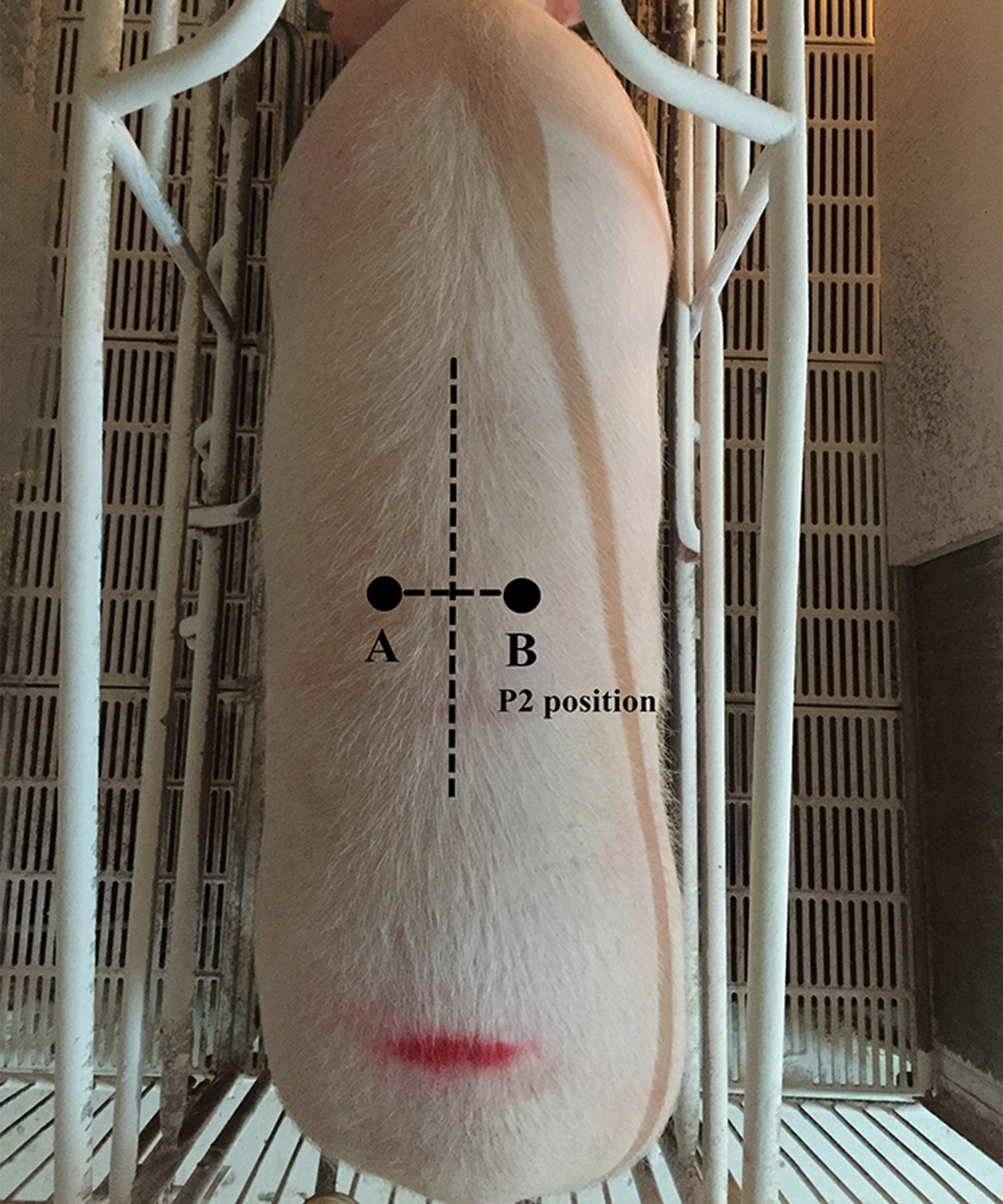

Supplement: Supplementary file 1 [file animals-09-00546-s001.zip › animals-542310 suppl/Figure S1.jpg]

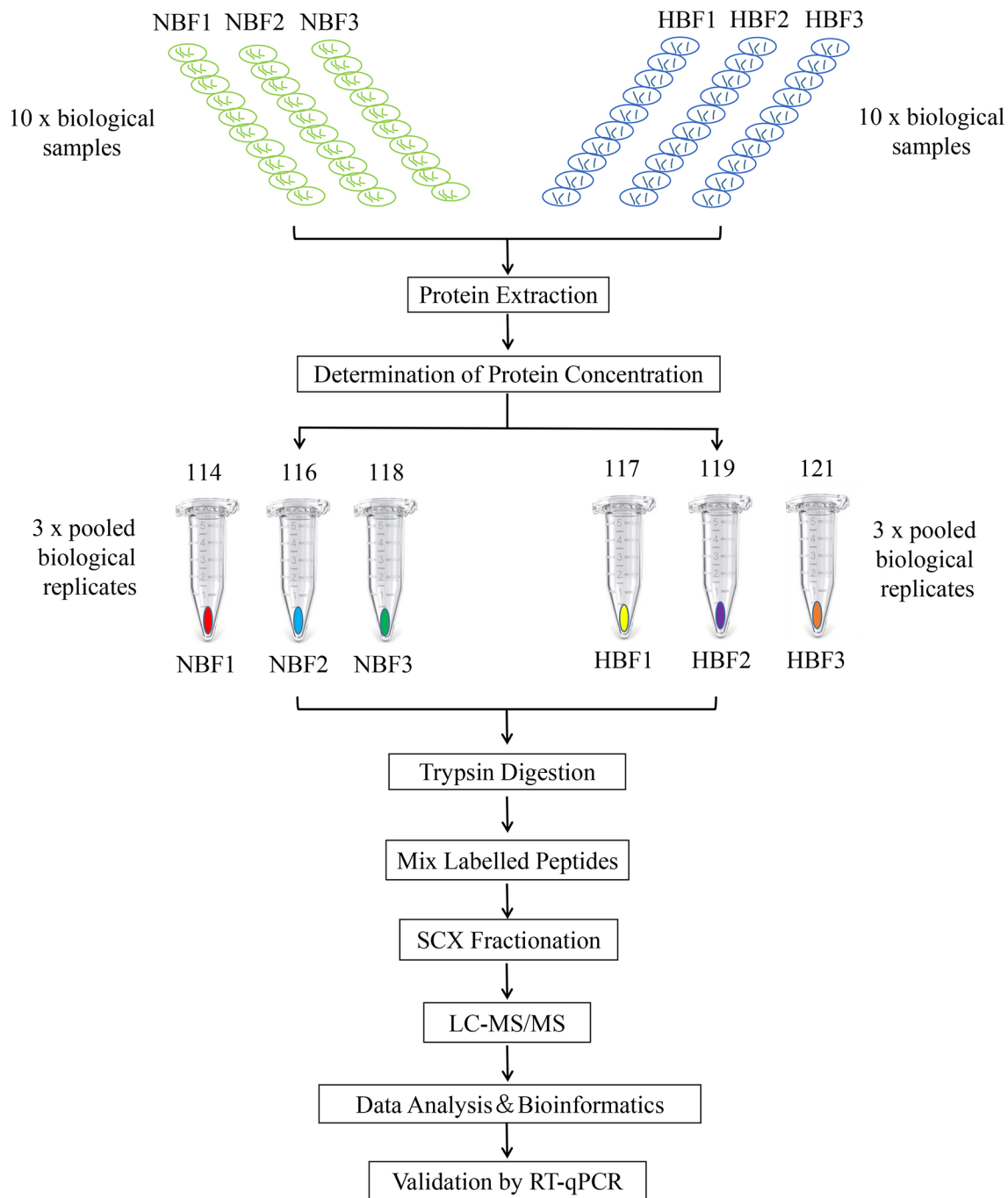

Supplement: Supplementary file 1 [file animals-09-00546-s001.zip › animals-542310 suppl/Figure S2.pdf]
