# Supplementary material for: The Effects of Maternal Obesity on Porcine Placental Efficiency and Proteome
Source: Animals (Basel). 2019 Aug 12;9(8):546. doi: 10.3390/ani9080546 (PMC6720507; doi:10.3390/ani9080546)
Supplement: Supplementary file 1 [file animals-09-00546-s001.zip › animals-542310 suppl/Table S1.doc]

Table S1. RT-qPCR primers.

| Gene Name | mRNA RefSeq ID | Sequence (5' - 3') | Product Size (bp) |
| --- | --- | --- | --- |
| GAPDH | NM_001206359.1 | F: TCGGAGTGAACGGATTTGGC | 189 |
|  |  | R: TGACAAGCTTCCCGTTCTCC |  |
| MMP9 | NM_001038004.1 | F: AGAACTTCTGGAGGTTCGACG | 184 |
|  |  | R: ACCTGATTCACCTCGTTCCG |  |
| ALB | NM_001005208.1 | F: ATGCTGCCAAGCTGCTGATA | 156 |
|  |  | R: AGGCGAGCTAATGACCATGC |  |
| PLG | NM_001044590.2 | F: TGCACACGAAGAGTACCACC | 222 |
|  |  | R: CCGTACGTGCCTTTGGTTTC |  |
| CPT1A | NM_001129805.1 | F: GGACCCCTGATGGTGAACAG | 162 |
|  |  | R: CGTGGATCCCAGGAGAATCG |  |
| TSPO | NM_213753.1 | F: CTCACGCAATGTCCTCGGAA | 121 |
|  |  | R: ATACCCCATGGCCGAGTAGA |  |
| LDHA | NM_001172363.2 | F: TGCCTGTATGGAGCGGAGTA | 179 |
|  |  | R: TGCCACAGATAGTCCAATGGC |  |
| OGDH | XM_021079061.1 | F: GAAGACCCACCTCACAGAGC | 216 |
|  |  | R: GCCTGATGCCTAGGTGAGC |  |
| GAPDHS | XM_021097231.1 | F: GATGGACTTGGAACGGTGGT | 212 |
|  |  | R: GCCATGGCCTCCTAAGGTTT |  |
| CS | NM_214276.1 | F: GCAGAGGGTATCCACCGAAC | 206 |
|  |  | R: AGCTCCGTGAACTGAGCATC |  |
| LDHB | NM_001113287.1 | F: ACGCTGCCGACTTTGTTCTC | 183 |
|  |  | R: GGCACATGCCATACCGACTT |  |
| ACO2 | NM_213954.1 | F: GGCTCCGGAATCATTCACCA | 193 |
|  |  | R: TCACACCAATCACCTTGGGG |  |
| HADHA | NM_213962.2 | F: AGGTAGAGGCGGTGATTCCA | 200 |
|  |  | R: TTCTACGGCCGAAGCAGTTG |  |
| ATP5B | XM_001929410.5 | F: AGGCTGGCTCAGAAGTGTCT | 203 |
|  |  | R: ATCCAAATGCGCAAAGGTGG |  |
| NDUFA12 | XM_003126715.4 | F: TCTTCAGGGCAAACGATGTGA | 111 |
|  |  | R: ATATAACCCACCGGTGACGG |  |
| COX5B | NM_001007517.1 | F: TGGGCTGCATCTGTGAAGAA | 166 |
|  |  | R: AGAAACTTGCCAGCGCATCT |  |
